# Increased in-hospital mortality following severe head injury in young children: results from a nationwide trauma registry

**DOI:** 10.1186/s40001-015-0159-8

**Published:** 2015-08-14

**Authors:** Philipp Lichte, Hagen Andruszkow, Miriam Kappe, Klemens Horst, Miguel Pishnamaz, Frank Hildebrand, Rolf Lefering, Hans-Christoph Pape, Philipp Kobbe, TraumaRegister DGU

**Affiliations:** Department of Orthopaedic Trauma Surgery, University Hospital Aachen, Pauwelsstraße 30, 52074 Aachen, Germany; Harald Tscherne Research Laboratory for Orthopedic Trauma, Department of Orthopaedic Trauma Surgery, University Hospital RWTH Aachen, Aachen, Germany; Institute for Research in Operative Medicine (IFOM), University of Witten/Herdecke, Ostmerheimer Str. 200, 51109 Cologne, Germany; Committee on Emergency Medicine, Intensive Care and Trauma Management, German Trauma Society (Sektion NIS), Cologne, Germany

## Abstract

**Background:**

In the current literature, the outcome of paediatric brain injury is controversially discussed. According to the majority of the studies, there seems to be a decreased mortality but worse recovery in paediatric, traumatic brain injury in comparison with adults. However, there is a lack of information concerning the differences in various stages of development in patients younger than 18 years. The aim of our study was to verify the in-hospital outcome of different paediatric age groups in comparison to adults with respect to the treatment strategy.

**Methods:**

We performed a retrospective analysis of the TraumaRegister DGU^®^ from 2002 to 2012. Inclusion criteria were an Abbreviated Injury Scale (AIS) head ≥3 points and an AIS ≤2 points of the remaining body regions. The collective was divided into different subgroups according to age (1–3, 4–6, 7–10, 11–14, 15–17) and an adult control group aged between 18 and 55 years. We descriptively analysed the endpoint rate of sepsis, multiple organ failure, and mortality. Additionally, the Glasgow Outcome Scale (GOS) at discharge was observed.

**Results:**

Overall, 1110 children and 6491 adult control patients were included. Comparing the rate of intubation on-scene, the rate of cranial CT scans, the rate of craniotomies, and the rate and length of intensive care treatment, we could only identify minor differences between the age groups. The treatment after discharge from hospital was markedly different due to a very low rate of in-patient rehabilitation treatment in children. On one hand, the rate of systemic complications, such as sepsis and multiple organ failure increased with increasing age. On the other hand, we found a significantly increased mortality in children younger than 7 years after very (AIS head = 5) severe brain injury. The in-hospital functional outcome in survivors, according to the GOS, was beneficial for younger children in comparison to adolescents and adults.

**Conclusions:**

We were unable to identify marked age-related differences in the therapeutic approach. Nevertheless, we were able to demonstrate marked differences of outcome. Children younger than 7 years significantly die more often due to direct impact of severe trauma. But if they survive, they seem to develop less systemic complications and profit from a better functional outcome.

## Background

Traumatic brain injury (TBI) is a major determinant of morbidity and mortality in trauma patients [1, 2]. In Germany, the overall incidence of TBI was reported to be approximately 332/100,000 inhabitants per year [3]. Despite a comparable incidence of TBI in children and adults, TBI represents the leading cause of death in children aged under 15 years [4].

In general, there seem to be significant differences between children and adults in the clinical course after TBI. In this context, children are supposed to be more capable to adapt to brain injury and demonstrate a lower mortality rate [5]. However, another study also indicated that there might be an inverse correlation between age and remaining neurological deficit [6]. Thus, information on a potential, favourable outcome following TBI in children or adolescents compared to adults seems to be controversial in the current literature. Different aspects might cause this lack of consent. Besides small sample sizes, an “aged-matched” prediction of morbidity and mortality might be further complicated by multiple interacting factors, such as overall injury severity, in-hospital treatment strategies, and rehabilitation measures [7]. Likewise, it remains unclear whether the treatment reality in the first phase after trauma differs in different age groups and whether treatment recommendations were followed.

Therefore, the aim of the present study was to analyse age-related differences in morbidity and mortality following trauma with leading TBI in a very large study cohort to avoid best the possible limitations of other studies.

## Methods

The TraumaRegister DGU^®^ of the German Trauma Society (Deutsche Gesellschaft für Unfallchirurgie, DGU) was founded in 1993. The aim of this multi-centre database is to establish an anonymous, standardized documentation of severely injured patients.

Data are collected prospectively in four consecutive time phases from the site of the accident until discharge from hospital: (A) pre-hospital phase, (B) emergency room and initial surgery, (C) intensive care unit, and (D) discharge. The documentation includes detailed information on demographics, injury pattern, comorbidities, pre- and in-hospital management, course on intensive care unit, and relevant laboratory findings including data on transfusion and outcome of each individual. The inclusion criterion is admission to hospital via emergency room with subsequent ICU/ICM care, or reaching the hospital with vital signs and die before admission to ICU.

The infrastructure for documentation, data management, and data analysis is provided by AUC—Academy for Trauma Surgery (AUC—Akademie der Unfallchirurgie GmbH), a company affiliated to the German Trauma Society. The scientific leadership is provided by the Committee on Emergency Medicine, Intensive Care and Trauma Management (Sektion NIS) of the German Trauma Society. The participating hospitals submit their data anonymously into a central database via a web-based application. Scientific data analysis is approved according to a peer review procedure established by Sektion NIS. The participating hospitals are primarily located in Germany (90 %), but a rising number of hospitals of other countries contribute data as well (at the moment from Austria, Belgium, China, Finland, Luxembourg, Slovenia, Switzerland, The Netherlands, and the United Arab Emirates). Currently, approx. 25,000 cases from more than 600 hospitals are entered into the database per year.

Participation in TraumaRegister DGU^®^ is voluntary. For hospitals associated with TraumaNetzwerk DGU^®^, however, the entry of at least a basic data set is obligatory for reasons of quality assurance.

The present study is in line with the publication guidelines of the TraumaRegister DGU^®^ and registered as TR-DGU project ID 2010-009.

### Inclusion criteria

The following patients were included in this study:

- Age at time of injury between 1 and 17 years (children) or 18–55 years (adult control group).
- Date of admission from January 1, 2002, to December 31, 2012.
- Direct transport from the scene of injury to the treating hospital.
- AIS head ≥3 points.
- AIS all other body regions ≤2 points.

The age limit (55 years) in the adult control group was set to have a coherent control group without geriatric patients.

Patients with incomplete data referring to demographic data and diagnoses as well as the Glasgow Outcome Scale (GOS) were excluded.

### Injury severity and distribution

Injury distribution was determined with the 2005 edition of the Abbreviated Injury Scale (AIS) and summarized in the Injury Severity Score (ISS) reflecting the overall injury severity [8]. Besides the established AIS score, the Glasgow Coma Scale (GCS) was used to describe the severity of TBI.

### Treatment modalities

The initial treatment was assessed by the documentation of intubation on-scene, intubation in the emergency room, and intubation on-scene in case of GCS ≤8 points. In the emergency room, the incidence of cranial CT scan or whole-body CT examination was analysed. Further treatment after discharge was documented in the categories relocation to another hospital, transfer to rehabilitation clinic, or return home.

### Clinical course and outcome assessment

The clinical course included duration of mechanical ventilation, the length of stay on the intensive care unit, and overall hospital stay. Mortality was analysed as main outcome. Complications during hospital treatment included sepsis and organ failure. The diagnosis of sepsis was made according to the criteria of the ACCP/SCCM consensus conference committee [9, 10]. Organ function status was evaluated according to the Sequential Organ Failure Assessment (SOFA) score [11]. With three or more points, an organ function was considered as failure, whereas multiple organ dysfunction syndrome (MODS) was defined as simultaneous failure of at least two organs. To assess the neurological outcome at hospital discharge, the Glasgow Outcome Scale (GOS) was used [12]:

- Death (GOS 1).
- Persistent vegetative state (GOS 2): patient exhibits no obvious cortical function.
- Severe disability (GOS 3: conscious but disabled): patient depends upon others for daily support due to mental or physical disability or both.
- Moderate disability (GOS 4: disabled but independent): patient is independent as far as daily life is concerned. The disabilities found include varying degrees of dysphasia, hemiparesis, or ataxia, as well as intellectual and memory deficits and personality changes.
- Good recovery (GOS 5): resumption of normal activities even though there may be minor neurological or psychological deficits.

### Study groups

To analyse age-dependent treatment strategies and in-hospital outcome, the included patients were divided into six subgroups referring to Andruszkow et al. [13]:

- Group I (“infants”): age 1–3 years.
- Group II (“pre-school-aged children”): age 4–6 years.
- Group III (“primary-school-aged children”): age 7–10 years.
- Group IV (“late childhood”): age 11–14 years.
- Group V (“adolescents”): age 15–17 years.
- Control Group VI (“adults”): age 18–55 years.

### Statistics

The data were analysed using the Statistical Package for the Social Sciences (SPSS; version 22; IBM Inc., Somers, NY, USA). Incidences are presented with counts or percentages, while continuous values are presented as mean ± standard deviation (SD).

Differences in mortality rates were calculated using the Chi-square test. Further formal statistical testing was avoided here due to the large number of variables considered. Furthermore, the five subgroups plus one control group would result in 15 pairwise test results, which do not seem to be justified. The large number of adult control individuals provides rather stable results in that subgroup. The paediatric subgroups with 100–400 cases each would allow to detect differences between 4 % (*n* = 400) and 8 % (*n* = 100) for categorical variables and less than 0.2 standard deviations for continuous variables.

## Results

Overall, 1110 patients younger than 17 years met the inclusion criteria. The adult control group consisted of 6491 patients. The average ISS of the entire group was 20.0 points (SD 14.2). Descriptive data of our population are shown in Table 1.

**Table 1:** Demographics

	Age 1–3 years	Age 4–6 years	Age 7–10 years	Age 11–14 years	Age 15–17 years	Adults
*n*	118	127	193	270	402	6491
Age [mean (SD)]	2.1 (0.8)	5.0 (0.8)	8.4 (1.1)	12.7 (1.1)	16.1 (0.8)	36.5 (11.7)
Male (%)	56.4	63.8	61.8	59.9	67.2	76.6
ISS (points)	20.3 (14.1)	18.6 (11.8)	17.0 (7.3)	18.1 (8.2)	19.4 (9.7)	20.2 (10.8)
GCS on-scene (points)	9.6 (4.5)	9.7 (4.6)	10.5 (4.3)	9.3 (4.5)	9.4 (4.7)	9.6 (4.7)

The distribution of the AIS head is demonstrated in Table 2. We were not able to detect marked differences between the severities of the brain injuries in the different age sets. In contrast, we were able to demonstrate crucial differences in the injury mechanisms (Table 3).

**Table 2:** Distribution of head injury severity according to AIS in age subgroups

Head injury severity	Age 1–3 years (%)	Age 4–6 years (%)	Age 7–10 years (%)	Age 11–14 years (%)	Age 15–17 years (%)	Adults (%)
AIS 3	32.2	40.9	45.6	42.6	38.9	37.1
AIS 4	42.4	39.4	39.9	35.6	35.9	32.3
AIS 5	20.3	16.5	14.0	21.1	23.5	28.3
AIS 6	5.1	3.1	0.5	0.7	1.7	2.4

**Table 3:** Type of accident related to age

	Age 1–3 years (%)	Age 4–6 years (%)	Age 7–10 years (%)	Age 11–14 years (%)	Age 15–17 years (%)	Adults (%)
Car	14.6	18.3	10.3	5.9	15.6	23.3
Motorcycle	1.0	0	0.5	2.4	23.7	9.3
Bicycle	0	8.3	19.0	27.3	15.1	12.4
Pedestrian	9.7	17.5	28.3	23.3	11.2	6.4
Low fall <3 m	27.2	22.5	14.7	15.8	11.2	20.6
High fall >3 m	29.1	15.0	17.4	9.9	8.1	14.3

**Fig. 1:**
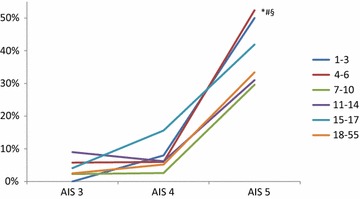
In-hospital mortality rate according to the AIS head. After very severe TBI, the mortality rate is significantly higher in children younger than 6 years compared to the older children (*p = 0.001), adolescents (^#^ *p* = 0.001), and also adults (^$^ *p* < 0.001)

**Fig. 2:**
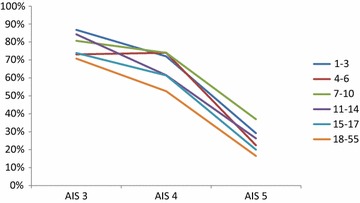
Low disability (GOS = 5) at discharge according to the AIS head. The percentage of children with good outcome is superior compared to those of adults and adolescents. The course of children between 4 and 6 years with an AIS 5 may be influenced by a low number of cases (*n* = 21)

Overall, the mortality rates showed differences between the different age groups (*p* = 0.004). Interestingly, we observed a higher mortality in children younger than 7 years in comparison to older children and adults (Table 4), especially after very severe TBI (AIS head = 5: 17.1 vs. 9.5 %, *p* = 0.001) (Fig. 1). On the other hand, in survivors, the functional outcome according to the GOS was better in younger patients: the overall percentage of patients with a low disability at discharge decreased with increasing age, and the rate of severe disabilities increased simultaneously. These differences were, again, most obvious in patients with very severe brain injury (AIS head = 5) (Fig. 2). Likewise, the length of ICU and hospital stay, as well as the days of mechanical ventilation accelerated with increasing age. Additionally, we evaluated a lower rate of systemic complications (sepsis, MOF) in children younger than 14 years in comparison to adolescents and adults.

**Table 4:** In-hospital outcome

	Age 1–3 years	Age 4–6 years	Age 7–10 years	Age 11–14 years	Age 15–17 years	Adults
Overall mortality (%)	17.8*	15.6*	7.0	10.4	9.3	13.7
Mortality AIS head >5 (%)	50.9*^#^	52,4*^#^	29.6	31.6	32.6	33.4
Low disability (GOS = 5) (%)	64.4	60.6	71.5	63.3	55.4	48.0
Severe disability (GOS 2 and 3) (%)	5.0	7.0	8.3	10.7	12.4	17.3
ICU (days, survivors)	6.0 (11.7)	4.9 (6.1)	5.5 (8.5)	5.5 (7.0)	8.3 (11.4)	8.2 (10.8)
Ventilator days (survivors)	2.5 (5.2)	2.6 (5.2)	2.7 (5.2)	3.3 (6.1)	5.2 (9.3)	5.1 (9.1)
Hospital stay (days, survivors)	13.2 (16.1)	11.6 (11.8)	12.1 (10.9)	11.7 (8.0)	17.7 (23.5)	17.0 (20.8)
Sepsis (%)	2.2	3.3	0.7	2.6	6.4	5.9
MOF (%)	16.1	18.2	17.2	15.0	19.3	23.5
SMR						

Data are presented as mean ± SD. Differences in mortality were analysed by Chi-square test: * *p* = 0.001 in comparison to children >6 years, ^#^ *p* < 0.001 in comparison to adults

Treatment modalities are shown in Table 5. The intubation rate on-scene was slightly increased in infants, especially in comparison to older children. In general, about 90 % of all unconscious patients (GCS <8) were intubated on-scene in all age groups except the infant group in which only 74.5 % of the unconscious were intubated. The cranial CT scan was used for diagnosis of TBI in 90 % of all patients. Craniotomy was performed in about 10 % of the patients in all age groups. Concerning the treatment after discharge, we identified marked differences: younger children more often returned home, whereas the rate of rehabilitation treatment increased with increasing age.

**Table 5:** Treatment modalities in the different groups of age

	Age 1–3 years	Age 4–6 years	Age 7–10 years	Age 11–14 years	Age 15–17 years	Adults
Intubation on-scene (%)	14.4	8.6	8.4	7.6	11.3	10.4
GCS ≤8 and intubation on-scene	74.5	87.5	89.4	94.1	88.4	87.7
Intubation at ER (%)	43.3	60.0	59.0	61.4	65.2	58.4
Cranial CT scan (%)	89.9	91.3	92.7	97.4	96.3	95.3
Whole-body CT scan (%)	32.5	34.1	40.3	34.9	50.0	56.2
Discharged home (%)	59.3	58.3	67.9	59.6	50.7	43.8
Craniotomy (%)	9.4	9.2	8.0	10.5	9.8	11.8
Transfer to a rehabilitation clinic (%)	15.3	15.7	16.6	21.1	27.6	29.0
Other hospital (%)	5.9	7.9	6.2	8.5	10.7	11.3

## Discussion

We descriptively analysed age-dependent differences concerning the treatment modalities and outcome of severe TBI. We focused on infants, children, and adolescents compared to adult trauma patients. Our main results can be summarized as follows:

- The initial treatment and diagnostics (rate of intubation and cranial CT scan) showed no relevant differences between the different age groups; however, children were less frequently transferred to rehabilitation centres.
- The mortality rate was significantly higher in children younger than 7 years, especially after severe TBI.
- Functional outcome after TBI was better for children in comparison to adolescents and adults, even after severe TBI.

### Demographics and injury patterns

Our study collective showed some special characteristics. For adult patients, the well-known higher proportion of males was found [14, 15], whereas in younger patients, a higher percentage of females was observed. These differences in the gender distribution were in line with our observation that the trauma mechanisms differ between children and adults and might be attributable to similar leisure behaviour and the missing influence of work accidents in children.

Overall, children were more often involved in pedestrian accidents in comparison to adults. Furthermore, infants were more often injured from falls, children between 11 and 14 from bicycle accidents, and adolescents from motorcycle accidents. These entities reflect the age-dependent changes of leisure behaviour and road traffic attendance. The high rate of severe traumatic brain injuries after bicycle accidents in the age between 11 and 14 might be included in the planning of campaigns for paediatric bicycle helmets. The differences in trauma mechanisms might at least be partially responsible for the wavelike formation of the brain injury severity (AIS head) with the lowest values in the age group between 7 and 10 years.

In our collective group, the overall ISS was relatively low due to the exclusion of all patients with an AIS >2 of the other body regions apart from the brain injury. In contrast to the AIS head, the distribution of the ISS over the different age groups was quite consistent.

### Clinical course and outcome

Apart from age-dependent differences, the outcome might be influenced by different treatment strategies in the various age groups. Due to the limited information in the registry, we focused on crucial steps, such as the intubation either on-scene or in-hospital, the implementation of a cranial CT scan in emergency room diagnostics, the length of intensive care treatment, and discharge modalities. Interestingly, the overall rate of intubation on-scene was higher in the infant group (Table 5). On the other hand, only 74.5 % of all unconscious (GCS ≤8) infants were intubated on-scene, whereas in all other groups, the rate was higher than 87.5 % and reached its maximum in the age of 11–14 years (94.5 %). Due to the design of the study, we were not able to prove the correct usage of the Paediatric Glasgow Coma Scale (PGCS) in younger children. Therefore, age-related limitations in the verbal answer might be a reason for difficulties in correct GCS documentation in the infant group [16]. Although von Elm et al. [17] concluded in their systematic review that no evidence for a better outcome after pre-hospital intubation in patients with TBI exists, another study described a benefit for children with TBI who had been intubated in the field [18].

CT diagnostics is a central part of the decision-making process in paediatric traumatic brain injuries because of the quick detection of surgically relevant lesions [19]. Especially in children, an increased risk for brain injury despite a normal head CT scan has been described [20]. Therefore, continuous neurological examination and, when in doubt, ICP measurement are recommended [20, 21]. Nevertheless, our data confirmed that about 90 % of all included patients received a cranial CT scan independent of their age. So, in daily practice, the cranial CT scan is used as an important tool to assess the need for neurosurgical intervention in children as well as in adults after head injury, as recommended [22]. Craniectomy was performed in about 10 % of all patients independent of age. Studies of craniotomies in children are rare [23]. Many neurosurgeons had been sceptical because of the risk to pay the decrease in mortality with an increase in severe disabilities [24]. On the other hand, a recent study has shown that decompressive craniectomy resulted in good recovery in all severely head-injured children in the study, suggesting that the procedure has an advantage over non-surgical treatment [25]. In a large study, Jagannathan et al. found about 65 % favourable outcomes in paediatric patients after craniectomy when followed for more than 5 years [26]. However, the rate of craniectomy in our study was comparable in all age groups.

In our analysis, we found no differences in the frequency of intensive care treatment, which is in line with other studies [15]. In contrast, the length of ICU and hospital treatment as well as mechanical ventilation in surviving patients was longer in children younger than 14 years.

After hospital treatment, children were more often discharged home and less often to rehabilitation centres. Possibly, there is a lack of paediatric rehabilitation units. This is in line with a lack of evidence in the literature for the need of early neurological rehabilitation in children [27], and special therapies for children are often still at the experimental stage [28].

Besides the injury’s severity as a critical predictor [29], age at time of injury has been suggested to have a significant impact on functional and cognitive recovery [29–32]. Several studies comparing outcome after TBI indicated that younger age is associated with worse recovery after injury compared to elder children [29, 33, 34]. In this context, it was concluded that young children might be more vulnerable to disruptions caused by TBI compared to older children, as their brain is more rapidly developing with considerable cognitive skill maturation [7, 31, 32, 35]. Our results are, at least partly, in contrast to these results: analysing outcome according to the severity of the TBI, children showed better results than adolescents and adults even in cases of very severe TBI (AIS = 5). Children between 4 and 14 years of age showed more often a good outcome (GOS = 5) in comparison to adults. Therefore, we believe that current clinical results [29–32] of deteriorated recovery for children have to be questioned at least in the early period after trauma.

Better recovery might be explained by the higher development potential of the young brain. Damages can be compensated by progressing development, reorganization, and myelinization processes [36]. In this context, a previous study showed that even children with the most severe brain injuries, who enter rehabilitation completely dependent for all daily activities, have the potential to make significant gains in functioning by the time of discharge and in the following few months [37]. In addition, further risk factors, such as pre-existing morbidities, deteriorate the outcome with increasing age [38] and might be responsible for the increasing rate of systemic complications, such as sepsis and MOF. On the other hand, the presence of TBI during a critical stage of brain development could have serious consequences which might be reflected by the higher mortality rate in infants.

### Mortality

Several studies approved the significant influence of age towards mortality after TBI. Our data showed a significantly increased mortality for children in the age group between 1 and 6 years and a slightly lower mortality in the age group between 7 and 14 years in comparison to adults. This distribution is similar to the study of Remmers et al. They observed a trend towards an increased mortality of pre-school-aged children and adolescents in comparison to adults, whereas children between 6 and 12 years showed a significant lower mortality rate [6]. In contrast, Luerssen et al. analysed 8,814 patients with head injuries and found a significantly lower mortality in the paediatric collective. Studies in adult patients showed a lower mortality in patients younger than 55 years compared with older patients [39]. Another study confirmed a significant influence of age on mortality with increased mortality in older patients [38]. Therefore, increasing age has been supposed to be an independent factor to predict mortality after TBI in adult patients [40]. In adults, younger age seems to be beneficial, whereas this is not transferable to children. Different reasons might be responsible for the nearly doubled death rate in infants in our collective. Infants have a relatively larger and heavier head with weak neck muscles, and a greater flexibility of cranial bones predisposing for diffuse brain injuries [29, 31, 33, 41, 42]. This may cause more severe brain injuries after comparable trauma, even if we could not demonstrate corresponding differences in brain injury severity according to the AIS head. Another explanation may be the difficulty to estimate the initial injury severity in small children. A wrong initial assessment may cause complications in the further therapy and may negatively influence the outcome [6, 42]. Several studies showed that children have a disposition to a more severe brain oedema after TBI, and are more vulnerable to increased pressure due to the relative tight cerebrospinal fluid spaces independently of initially comparable injury severity. Additionally, they are more sensitive for hypoxia and hypercapnia [6, 28, 43].

### Limitations

For all of the advantages of our large study collective, some limitations of our study deserve further comments. Although there is an Internet-based integrated plausibility check, data quality and completeness of the documented parameters are usually lower in registries as compared to prospective clinical studies. Due to the large difference in the number of patients of the children subgroup in comparison to the control group and the large number of variables, differentiated statistical analysis was not reasonably feasible. Therefore, we focussed on mortality for statistic analysis. Analysing outcome differences, our study does not differentiate between the severity and length of MOF and sepsis.

## Conclusion

In conclusion, we observed age-related differences in the mortality rate and functional outcome after severe traumatic brain injury despite similar strategies in the first period of treatment. The mortality rate for children below the age of 7 and with a severe brain injury (AIS 5) was significantly higher than in the older groups. On the other hand, in survivors of a severe brain injury, we could observe a higher rate of good functional outcome in younger children.
